# p53 missense but not truncation mutations are associated with low levels of *p21^CIP1/WAF1^* mRNA expression in primary human sarcomas

**DOI:** 10.1054/bjoc.2001.1844

**Published:** 2001-06

**Authors:** S Mousses, N Gokgoz, J S Wunder, H Ozcelik, S Bull, R S Bell, I L Andrulis

**Affiliations:** Samuel Lunenfeld Research Institute; Departments of Laboratory Medicine and Pathobiology; University Muscoskeletal Oncology Unit, Mount Sinai Hospital, 600 University Ave, Toronto, Ontario, M59 1XS; Departments of Molecular and Medical Genetics, University of Toronto, Ontario, M5S 1A8, Canada

**Keywords:** sarcomas;*p21^CIP1/WAF1^* expression;*p53* mutations

## Abstract

Many growth-suppressing signals converge to control the levels of the *CDK* inhibitor p21^CIP1/WAF1^. Some human cancers exhibit low levels of expression of p21^CIP1/WAF1^and mutations in *p53* have been implicated in this down-regulation. To evaluate whether the presence of *p53* mutations was related to the *in vivo* expression of *p21^CIP1/WAF1^* mRNA in sarcomas we measured the *p21^CIP1/WAF1^* mRNA levels for a group of 71 primary bone and soft tissue tumours with known *p53* status. As expected, most tumours with *p53* mutations expressed low levels of *p21^CIP1/WAF1^* mRNA. However, we identified a group of tumours with *p53* gene mutations that exhibited normal or higher levels of *p21^CIP1/WAF1^* mRNA. The *p53* mutations in the latter group were not the common missense mutations in exons 4–9, but were predominantly nonsense mutations predicted to result in truncation of the p53 protein. The results of this study suggest that different types of *p53* mutations can have different effects on the expression of downstream genes such as *p21^CIP1/WAF1^* in human sarcomas. © 2001 Cancer Research Campaign http://www.bjcancer.com


*British Journal of Cancer* (2001) **84**(12), 1635–1639
© 2001 Cancer Research Campaign
doi: 10.1054/ bjoc.2001.1844, available online at http://www.idealibrary.com on IDEAL®

http://www.bjcancer.com

### p53 missense but not truncation mutations are associated with low levels of *p21^CIP1/WAF1^* mRNA expression in primary human sarcomas

**S Mousses[1,2,*], N Gokgoz[1,*], JS Wunder[1,3], H Ozcelik[1,2], S Bull[1], RS Bell[1,3] and IL Andrulis[1,2,4]**

[1]Samuel Lunenfeld Research Institute; [2]Departments of Laboratory Medicine and Pathobiology; [3]University Muscoskeletal Oncology Unit, Mount Sinai Hospital, 600 University Ave, Toronto, Ontario, M59 1XS; [4]Departments of Molecular and Medical Genetics, University of Toronto, Ontario, M5S 1A8, Canada

**Summary** Many growth-suppressing signals converge to control the levels of the *CDK* inhibitor $p21^{CIP1/WAF1}$. Some human cancers exhibit low levels of expression of $p21^{CIP1/WAF1}$ and mutations in *p53* have been implicated in this down-regulation. To evaluate whether the presence of *p53* mutations was related to the *in vivo* expression of $p21^{CIP1/WAF1}$ mRNA in sarcomas we measured the $p21^{CIP1/WAF1}$ mRNA levels for a group of 71 primary bone and soft tissue tumours with known *p53* status. As expected, most tumours with *p53* mutations expressed low levels of $p21^{CIP1/WAF1}$ mRNA. However, we identified a group of tumours with *p53* gene mutations that exhibited normal or higher levels of $p21^{CIP1/WAF1}$ mRNA. The *p53* mutations in the latter group were not the common missense mutations in exons 4–9, but were predominantly nonsense mutations predicted to result in truncation of the p53 protein. The results of this study suggest that different types of *p53* mutations can have different effects on the expression of downstream genes such as $p21^{CIP1/WAF1}$ in human sarcomas. © 2001 Cancer Research Campaign http://www.bjcancer.com

**Keywords**: sarcomas; $p21^{CIP1/WAF1}$ expression; *p53* mutations

Sarcomas are malignant neoplasms of mesenchymal origin which, like other malignancies, are characterized by unbalanced cellular proliferation and genomic instability. One hypothesis, which may help explain their neoplastic phenotype, is that they arise as a consequence of a cell cycle checkpoint defect (Hartwell, 1992; Paulovich et al, 1997). Support for this hypothesis comes from the observation that many of the molecules that regulate cell cycle checkpoints in eukaryotic cells are direct targets for deregulation in human cancers. p53 protein for example is a critical component of a pathway that regulates the cell cycle at the G1/S phase in response to DNA damage (Waldman et al, 1995; Levine, 1997). In addition to p53 protein, other molecules in this pathway have been shown to be altered in human cancer cells (Hollstein et al, 1991; Hartwell, 1992; Kastan et al, 1992; Waldman et al, 1995; Levine, 1997). A number of groups, including our own (Mousses et al, 1996), have reported that the *p53* gene is frequently targeted for deregulation in bone and soft tissue sarcomas. The retinoblastoma (*RB*) gene product, which functions downstream of p53 is also commonly altered in sarcomas (Reissmann et al, 1989; Wunder et al, 1991). Interestingly, both *p53* and RB germ-line mutations predispose individuals to the development of sarcomas (Malkin et al, 1990; Bookstein and Lee, 1991). Other downstream genes, including *MDM*-2, which regulates p53 function (Finaly, 1993) and p53 protein expression, as well as *CDK4*, have been shown to be over-expressed and amplified in certain sarcomas (Khatib et al, 1993; Cordon-Cardo et al, 1994; Wunder et al, 1999).

A recently identified member of the p53-dependent DNA damage response pathway is the CDK inhibitor $p21^{CIP1/WAF1}$ (El-Deiry et al, 1993; Harper et al, 1993; Xiong et al, 1993; Noda et al, 1994). In the presence of DNA damage, p53 leads to the transcriptional activation of $p21^{CIP1/WAF1}$, via a responsive element in its promoter, and increased $p21^{CIP1/WAF1}$ expression then mediates a cell cycle arrest (El-Deiry et al, 1993, 1994). Increased levels of $p21^{CIP1/WAF1}$ cause inhibition of CDK/cyclin complexes and cell cycle arrest at the G1/S phase (Harper et al, 1993). Since its initial discovery, $p21^{CIP1/WAF1}$ has been observed to be regulated by both p53-dependent and independent mechanisms (El-Deiry et al, 1994; Macleod et al, 1995). Elevated $p21^{CIP1/WAF1}$ levels affect a wide range of cellular processes besides cell cycle checkpoints, including senescence and differentiation (El-Deiry et al, 1994; Macleod et al, 1995). There is mounting evidence that $p21^{CIP1/WAF1}$ is also targeted for deregulation in human cancer cells (El-Deiry et al, 1995; Ozcelik et al, 1995; Matsushita et al, 1996; Hui et al, 1997). Despite the fact that somatic mutations are not a common mechanism of inactivating $p21^{CIP1/WAF1}$ function (Shiohara et al, 1994; Koopmann et al, 1995; Mousses et al, 1995; Sun et al, 1995; Watanabe et al, 1995; Wan et al, 1996), cancer cells often have very low levels of $p21^{CIP1/WAF1}$ in comparison to their normal counterparts (El-Deiry et al, 1995; Ozcelik et al, 1995; Matsushita et al, 1996; Hui et al, 1997). Furthermore, $p21^{CIP1/WAF1}$, which is normally found in a complex with a cyclin, cdk and PCNA, is often missing from this quaternary complex in cancer cell lines (Xiong et al, 1993). The in vivo mechanisms leading to p21 deregulation and the consequences on the phenotype of the cancer cell are largely unknown and the topic of intense investigation.

Mutation of *p53* gene has been shown to inactivate its ability to transcriptionally activate downstream genes (Chen et al, 1993; Levine, 1997), including $p21^{CIP1/WAF1}$, leading to loss of cell cycle control (Li et al, 1994). In support of this, several studies have

*Received 28 April 2000*
*Revised 12 March 2001*
*Accepted 20 March 2001*

*Correspondence to:* IL Andrulis

* These authors contributed equally to this work.

shown that tumours with *p53* mutations tend to have lower steady state levels of $p21^{CIP1/WAF1}$ expression than tumours without such mutations (Ozcelik et al, 1995; Matsushida et al, 1996; Hui et al, 1997). We have investigated this association in human breast cancer and observed that tumours with *p53* mutations had a significantly lower distribution of $p21^{CIP1/WAF1}$ mRNA levels compared to tumours without detectable *p53* mutation (Ozcelik et al, 1995).

In a study of bone and soft tissue sarcomas, we had previously found that 15 to 30% of sarcomas have p53 mutations (Mousses et al, 1996). In this report we examined whether $p21^{CIP1/WAF1}$ gene expression was deregulated in primary human sarcoma specimens. To this end, we determined the level of $p21^{CIP1/WAF1}$ mRNA in a group of 71 bone and soft tissue sarcoma specimens and compared $p21^{CIP1/WAF1}$ mRNA expression with *p53* mutational status.

### MATERIAL AND METHODS

### Clinical data and tumour samples

Specimens were obtained from 71 patients with biopsy proven bone or soft tissue sarcoma. For each case, a surgically obtained tumour sample was chosen by a pathologist with the aid of frozen histologic analysis to determine that only viable tumour without contamination by surrounding normal tissue was chosen. Specimens were immediately snap frozen and stored at –70˚C. RNA was extracted by the guanidinium thiocyanate-cesium chloride gradient method (Chomczynski and Sacchi, 1987).

There were 51 bone sarcomas (mostly osteosarcoma and chondrosarcoma) and 20 soft tissue sarcomas (mostly liposarcoma and malignant fibrous histiocytoma). Most patients presented with a localized tumour (n = 66), while 5 had metastases present at the time of diagnosis. Treatment information was available for most patients. For 31 patients, the biopsy was followed by surgical excision of the primary tumour. However, 30 patients received neoadjuvant treatment prior to surgical resection. In general, preoperative treatment for patients with high-grade bone sarcomas consisted of adriamycin-based chemotherapy, while soft tissue sarcomas received 50 Gray local radiation. Tumour excision was usually performed 3–4 weeks following the completion of preoperative therapy.

### Analysis of mRNA expression by quantitative RT-PCR

cDNA synthesis and quantitative RT-PCR assays of $p21^{CIP1/WAF1}$ mRNA were performed essentially as described in Ozcelik et al (1995). CDNA was reverse-transcribed from 100 ng of total cellular RNA with random hexadeoxynucleotide primers and Moloney murine leukaemia reverse transcriptase (MMLV-RT). A fragment of $p21^{CIP1/WAF1}$ (5′-AAGACCATGTGGACCTGTCA-3′ and 5′-GGCTTCCTCTTGGAGAAGAT-3′ length of PCR product: 170 bp), and an internal control gene phosphoglycerate kinase (*PGK*) (5′-CAGTTTGGAGCTCCTGGAAG-3′ and 5′-TGCAAATCCAGGGTGCAG TG-3′ length of PCR product: 247 bp), were amplified in the same PCR reaction using Taq DNA polymerase. *PGK* was chosen as an internal control to account for variations in the quality of mRNA originally used and for variations in the PCR kinetics. Reactions were conducted over a range of cycles (22–28 cycles) in order to allow quantitative evaluation within the logarithmic phase of the PCR reaction.The PCR products were separated by PAGE, and quantified by laser densitometry (Molecular Dynamics). The expression of $p21^{CIPI/WAFI}$ was calculated as the ratio of the intensity of the PCR fragments for $p21^{CIPI/WAFI}$ to *PGK* (averaged over 2 cycle points within the logarithmic phase) relative to the breast cancer cell line, MCF-7.

### Statistical analysis

Comparisons in p21 levels among treatment groups and among p53 groups were conducted by exact *P* value calculations using Fisher's exact test for 2 by 2 tables and its generalization for 2 by 3 tables. Exact 95% confidence intervals (CI) were constructed to assess the precision of the observed differences in proportions.

### RESULTS

### Expression of $p21^{CIPI/WAFI}$ mRNA in sarcomas

The steady state levels of $p21^{CIPI/WAFI}$ mRNA were quantified by RT-PCR in 71 bone and soft-tissue tumours. Relative to an internal control gene, *PGK*, the levels of $p21^{CIPI/WAFI}$ expression were found to vary among tumours over a 10-fold range (Figure 1). An osteoblast cell line used as a normal control exhibited a relative level of expression of 0.72 (data not shown), and served to distinguish between tumours with low and high p21 expression. A number of sarcomas had very low levels of $p21^{CIPI/WAFI}$ expression when compared to the normal osteoblast cell line.

### p53 mutations and p21 mRNA expression

We had previously detected *p53* mutations in 29 of the 71 sarcoma specimens (Mousses et al, 1996; Gokgoz et al, submitted). When the *p53* status was compared with the $p21^{CIPI/WAFI}$ mRNA expression level (Figure 1) we found that in tumours with and without detectable *p53* mutations, the levels of $p21^{CIPI/WAFI}$ mRNA were distributed throughout the entire range of expression. There was no significant difference in p21 levels between tumours with and without *p53* mutations ($P$ = 0.76). The majority of tumours with

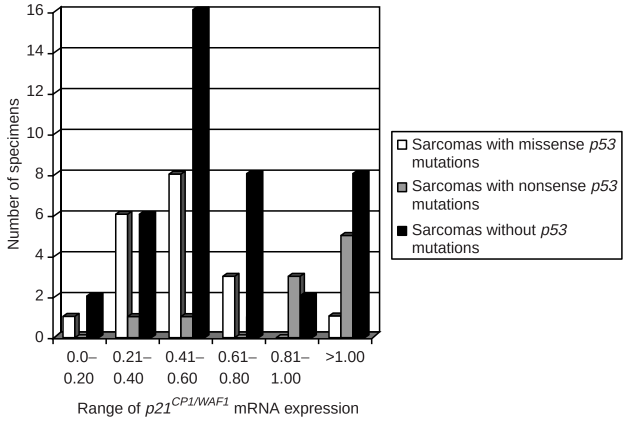

**Figure 1** Distribution of mRNA $p21^{CIP1/WAF1}$ expression in sarcomas of different *p53* status. A fragment of $p21^{CIP1/WAF1}$ and an internal control gene phosphoglycerate kinase (*PGK*) were amplified simultaneously. *PGK* was chosen as an internal control to account for variations in the quality of mRNA originally used and for variations in the PCR kinetics. Reactions were conducted over a range of cycles (22–28 cycles) in order to allow quantitative evaluation within the logarithmic phase of the PCR reaction. The PCR products were separated by PAGE, and quantified by laser densitometry (Molecular Dynamics). The expression of $p21^{CIP1/WAF1}$ was calculated as the ratio of the intensity of the PCR fragments for $p21^{CIP1/WAF1}$ to *PGK* relative to the tumour cell line MCF-7. The distribution of $p21^{C1P1/WAF1}$ mRNA levels is shown for sarcomas with and without detectable *p53* mutations

*© 2001 Cancer Research Campaign*

mutant *p53* (19 of 29, 66%) and wild-type *p53* (29 of 42, 69%) expressed low levels of $p21^{CIPI/WAFI}$ mRNA relative to the normal osteoblast cell line. However, there were 10 tumours with *p53* mutations that exhibited normal or higher levels of $p21^{CIPI/WAFI}$ mRNA.

### Association of missense mutations with low levels of $p21^{CIPI/WAFI}$ mRNA

To explore whether the specific type of *p53* mutation was associated with the level of expression of $p21^{CIPI/WAFI}$ mRNA, we compared the type of *p53* mutation (missense vs. other) in tumours with low levels versus tumours with high $p21^{CIPI/WAFI}$ levels. Of the 19 tumours with *p53* mutations that expressed low levels of $p21^{CIPI/WAFI}$, 17 had missense mutations (Table 1). In contrast, only 2 of the 10 tumours with *p53* mutations and high levels of $p21^{CIPI/WAFI}$ mRNA exhibited missense alterations (in codons 244 and 245). The majority of tumours with high $p21^{CIPI/WAFI}$ levels (8 of 10) exhibited *p53* mutations leading to stop codons that would potentially produce truncated forms of the p53 protein. The Fisher's exact test indicated a statistically significant association of missense mutations with low levels of $p21^{CIPI/WAFI}$ mRNA ($P = 0.0004$).

### The effect of pre-operative treatment on *p21* expression

We did not detect an association between $p21^{CIPI/WAFI}$ mRNA level and tumour grade, presentation with a primary versus metastatic lesion or locally recurrent tumour, or bone versus soft tissue sarcoma (Fisher's exact test $P > 0.05$). We did, however, observe a statistically significant association between $p21^{CIPI/WAFI}$ mRNA level and treatment. Treatment information was available for 61 of the patients in this study. 30 patients received either preoperative chemotherapy or radiotherapy prior to tumour resection; whereas the other 31 patients did not. In the untreated group, 22.6% (7 of 31) of tumours expressed high levels of $p21^{CIPI/WAFI}$ mRNA whereas 50% (15 of 30) of the treated group had elevated $p21^{CIPI/WAFI}$ mRNA (exact $P = 0.034$; difference in proportions = 27 (percentage units) with exact 95% CI of (1, 52)). A similar treatment difference was observed when the analysis was limited to the group of tumours with wild-type *p53*, although it did not attain conventional statistical significance (exact $P = 0.075$, difference = 32 with exact 95% CI of (–2, 65)) (Table 2).

In the untreated group, significant differences in $p21^{CIPI/WAFI}$ mRNA expression were observed among the 3 *p53* groups (exact $P = 0.003$). None of 9 tumours (0%) with missense mutations and 4 of 19 tumours (21%) without *p53* mutations had high $p21^{CIPI/WAFI}$ levels; whereas all 3 of the tumours with nonsense mutations (100%) expressed high levels of $p21^{CIPI/WAFI}$ mRNA. A similar pattern of differences was observed in the treated group (25, 53, 71% with high $p21^{CIPI/WAFI}$ in missense, wild-type, and nonsense groups, respectively), but was not statistically significant (exact $P = 0.25$). Although the $p21^{CIPI/WAFI}$ differences between missense and nonsense groups were greater in the untreated group than in the treated group (exact *P* values of 0.005 and 0.13, respectively, with $P = 0.001$ in the combined group), the 95% CIs for the 2 groups were wide and had substantial overlap.

**Table 1** *p53* mutations and $p21^{CIP1/WAF1}$ expression

| Type of sarcoma | p53 Codon | Mutation | $p21^{CIP1/WAF1}$ mRNA |
|---|---|---|---|
| Osteosarcoma | 337 | Arg > Cys | 0.19 |
| Osteosarcoma | intron 8 | splicing | 0.23 |
| Rhabdomyosarcoma | 132 | Lys > Glu | 0.26 |
| Osteosarcoma | Immunohistochemistry positive | [a]not done | 0.32 |
| Osteosarcoma | 248 | Arg > Gln | 0.33 |
| Osteosarcoma | 248 | Arg > Gln | 0.35 |
| Osteosarcoma | 248 | Arg > Gln | 0.35 |
| Osteosarcoma | Immunohistochemistry positive | [a]not done | 0.39 |
| Osteosarcoma | 273 | Arg > His | 0.42 |
| Osteosarcoma | 47 | Pro > Leu | 0.42 |
| Osteosarcoma | 281 | Asp > His | 0.45 |
| Liposarcoma | 276 | Ala > Pro | 0.46 |
| Osteosarcoma | 256 | Thr > Ser | 0.49 |
| Osteosarcoma | 237 | Met > Ile | 0.51 |
| Osteosarcoma | 202–206 | in frame deletion | 0.51 |
| Osteosarcoma | 220 | Tyr > Cys | 0.59 |
| Osteosarcoma | 250 | Pro > Leu | 0.59 |
| Osteosarcoma | 273 | Arg > His | 0.63 |
| Osteosarcoma | 242 | Cys > Tyr | 0.66 |
| Liposarcoma | 244 | Gly > Ser | 0.79 |
| Osteosarcoma | 342 | Arg > Stop | 0.81 |
| Osteosarcoma | 107/108 | in frame insertion | 0.84 |
| Osteosarcoma | 204 | Glu > Stop | 1.00 |
| Osteosarcoma | intron 5 | splicing | 1.07 |
| Osteosarcoma | 221 | Glu > Stop | 1.09 |
| Liposarcoma | 213 | Arg > Stop | 1.10 |
| Osteosarcoma | 43 | Leu > Stop | 1.11 |
| Osteosarcoma | 43 | Leu > Stop | 1.12 |
| Osteosarcoma | 245 | Gly > Ser | 1.45 |

[a]For two samples, *p53* mutations were not determined by DNA analysis but these samples exhibited strong immunohistochemical positive staining (Mousses et al, 1996).

*© 2001 Cancer Research Campaign* 

**Table 2** Association of *p21*$^{CIP1/WAF1}$ status in untreated and treated patients

| | | *p53* status | | |
|---|---|---|---|---|
| | | MS *p53* | NS *p53* | WT *p53* |
| Untreated | *p21* low | 9 | 0 | 15 |
| | *p21* high | 0 | 3 | 4 |
| Treated | *p21* low | 6 | 2 | 7 |
| | *p21* high | 2 | 5 | 8 |

MS = missense p53 mutations; NS = nonsense p53 mutations;
WT = wild-type *p53*.

### DISCUSSION

Down-regulation of *p21*$^{CIPI/WAFI}$ gene expression has been shown to occur in a wide variety of human cancer cell lines. Often this down-regulation is a result of a *p53* mutation, which leads to a loss of transcriptional activation of the *p21*$^{CIPI/WAFI}$ gene. Consequently many tumours with *p53* mutations have very low levels of *p21*$^{CIPI/WAFI}$. In vivo validation of genetic interactions that have been established in vitro, however, requires the use of tumour tissue. In this study, we investigated 2 genetic events, *p53* mutational status and expression of *p21*$^{CIPI/WAFI}$ mRNA, to determine whether they are associated in primary human sarcomas.

The steady state levels of *p21*$^{CIPI/WAFI}$ mRNA were quantified by RT-PCR in 71 bone and soft tissue sarcomas. The expression of *p21*$^{CIPI/WAFI}$ mRNA was found to vary among tumours over a 10-fold range and it was observed that some, but not all, of the sarcomas had relatively low levels when compared to the normal osteoblast cell line.

We found, as expected, that most tumours with *p53* mutations expressed low levels of *p21*$^{CIPI/WAFI}$ mRNA. However, we identified a group of tumours with *p53* gene mutations that exhibited normal or higher levels of *p21*$^{CIPI/WAFI}$ mRNA. The *p53* mutations in the latter group were not the common missense mutations in exons 4–9, but were predominantly nonsense mutations predicted to result in truncation of the p53 protein. The results of this study suggest that different types of *p53* mutations can have different effects on the expression of downstream genes such as *p21*$^{CIPI/WAFI}$ in human sarcomas.

Our observation of sarcomas with *p53* gene mutations that expressed normal or high levels of *p21*$^{CIPI/WAFI}$ mRNA are in contrast to those we previously reported in breast cancer. We had found that with very few exceptions, breast cancers with *p53* mutations had lower levels of *p21*$^{CIPI/WAFI}$ mRNA than breast cancers with wild-type *p53* (Ozcelik et al, 1995). It is possible that in addition to *p53* mutations, there may be other transcription factors, which influence the expression of *p21*$^{CIPI/WAFI}$, and are tissue specific for sarcomas. This would explain why many of the tumours with apparently low levels of *p21*$^{CIPI/WAFI}$ mRNA do not have detectable *p53* mutations. Furthermore, it may be that in breast cancer, most of the defects on p53 function are accomplished directly by *p53* gene mutation, whereas in sarcomas there may be more indirect p53 function defects. For example MDM2, which binds to and inactivates p53 and regulates the expression of p53 protein, has been shown to be amplified in some sarcomas (Khatib et al, 1993; Cordon-Cardo et al, 1994; Wunder et al, 1999).

Another possibility is that different types of *p53* mutations have various effects on the ability of p53 to transcriptionally induce the *p21*$^{CIPI/WAFI}$ promoter. For example, it has been shown in vitro that not all *p53* mutations lead to the same degree of loss of expression, and that some mutations may produce a gain of function (Chen et al, 1993; Ditmer et al, 1993).

A study by Taubert et al reported that missense *p53* mutations were associated with a worse prognosis for patients with sarcomas when compared to non-sense mutations (Taubert et al, 1996). It also has been observed that *p53* mutants without a functional tetramerization domain are not oncogenic (Chene and Bechter, 1999). Our data are compatible with these findings since we found that tumours with missense mutations generally expressed lower *p21*$^{CIPI/WAFI}$ mRNA levels than those tumours with truncation-type mutations. Higher levels of *p21*$^{CIPI/WAFI}$ mRNA are more representative of the normal cellular expression pattern.

There was no association between *p21*$^{CIPI/WAFI}$ mRNA level and tumour grade, stage or type of sarcoma, however, there was a significant association of high *p21*$^{CIPI/WAFI}$ levels with tumours which had received preoperative treatment with either chemotherapy or radiation. Although the differences in *p21*$^{CIPI/WAFI}$ expression according to *p53* status in the treated group were not significant at the 5% level, it should be recognized that only very large differences could be detected as significant in a sample of this size. Similarly, although the differences between missense and nonsense mutation were smaller in the treated group than in the untreated group, tests for homogeneity had low power to determine whether treatment modified the association between *p21*$^{CIPI/WAFI}$ mRNA level and *p53*.

In this study, we found that in primary human sarcomas, missense *p53* mutations were associated with lower levels of *p21*$^{CIPI/WAFI}$ expression; whereas *p53* truncation mutations were associated with higher expression. These observations warrant further investigation into the effects of various types of *p53* mutations on the transcriptional activation of the *p21*$^{CIPI/WAFI}$ promoter and on the expression of other downstream genes.

### ACKNOWLEDGEMENTS

We thank the Department of Pathology, Mount Sinai Hospital and orthopaedic surgeons, M Rock, R Grimer, J Healey, C Conrad III and C Beauchamp, for specimens. This work was supported by grants from National Cancer Institute of Canada (to ILA, RSB and JSW).

### REFERENCES

Bookstein R and Lee WH (1991) Molecular genetics of the retinoblastoma suppressor gene. *Crit Rev Oncog* **2**: 211–227

Chen J-Y, Funk WD, Woodring EW, Shay JW and Minna JD (1993) Heterogeneity of transcriptional activity of mutant *p53* protein and *p53* DNA target sequences. *Oncogene* **8**: 2159–2166

Chene P and Bechter E (1999) p53 mutants without a functional tetramerisation domain are not oncogenic. *J Mol Biol* **286**: 1269–1274

Chomczynski P and Sacchi N (1987) Single-step method of RNA isolation by acid guanidinium thiocyanate-phenol-chloroform. *Anal Biochem* **162**: 156–159

Cordon-Cardo C, Latres E, Drobnjak M, Oliva MR, Pollack D, Woodruff JM, Marechal V, Chen J, Brennan MF and Levine AJ (1994) Molecular abnormalities of mdm2 and *p53* genes in adult soft tissue sarcomas. *Cancer Res* **54**: 794–799

Dittmer D, Pati S, Zambetti G, Chu S, Teresky AK, Moore M, Finlay C and Levine AJ (1993) Molecular abnormalities of mdm2 and *p53* genes in adult soft tissue sarcomas. *Nature Genet* **4**: 42–45.

El-Deiry WS, Tokino T, Velculescu VE, Levy DB, Parsons R, Trent JM, Lin D, Mercer WE, Kinzler KW and Vogelstein B (1993) WAF1, a potential mediator of *p53* tumour suppressor. *Cell* **75**: 817–825.

 © 2001 Cancer Research Campaign

El-Deiry WS, Harper JW, O'Connor PM, Velculescu VE, Canman CE and Jackman J (1994) *WAF1/CIP1* is induced in *p53*-mediated $G_1$ arrest and apoptosis. *Cancer Res* **54**: 1169–1174.

El-Deiry WS, Tokino T, Waldman T, Oliner JD, Velculescu VE, Burrell M, Hill DE, Healy E, Rees JL and Hamilton SR (1995) Topological control of p21WAF1/CIP1 expression in normal and neoplastic tissues. *Cancer Res* **55**: 2910–2919.

Finlay CA (1993) The mdm-2 oncogene can overcome wild-type *p53* suppression of transformed cell growth. *Mol Cell Biol* **13**: 301–306.

Gokgoz N, Mousses S, Wunder JS, Eskandarian S, Bell RS and Andrulis IL. Mutations in the p53 gene are an early event in human osteosarcoma progression. (submitted)

Hartwell LH (1992) Defects in a cell cycle checkpoint may be responsible for the genomic instability of cancer cells. *Cell* **71**: 543–546.

Harper JW, Adami GR, Wei N, Keyomarsi K and Elledge J (1993) The p21 Cdk-interacting protein Cip1 is a potent inhibitor of G1 cyclin-dependent kinases. *Cell* **75**: 805–816.

Hollstein M, Sidransky D, Vogelstein B and Harris CC (1991) *p53* mutations in human cancer. *Science* **253**: 49–53.

Hui AM, Kanai Y, Sakamoto M, Tsuda H and Hirohashi S (1997) Reduced p21 (WAF1/CIP1) expression and *p53* mutation in hepatocellular carcinomas. *Hepatology* **25**: 575–579.

Kastan MB, Zhan Q, el-Deiry WS, Carrier F, Jacks T, Walsh WV, Plunkett BS, Vogelstein B and Fornace AJ Jr (1992) A mammalian cell cycle checkpoint pathway utilizing p53 and GADD45 is defective in ataxia-telangiectasia. *Cell* **71**: 587–597.

Khatib ZA, Matsushime H, Valentine M, Shapiro DN, Sherr CJ and Look AT (1993) Coamplification of the CDK4 gene with MDM2 and GLI in human sarcomas. *Cancer Res* **53**: 5535–5541.

Koopmann J, Maintz D, Schild S, Schramm J, Louis DN, Wiestler OD and von Deimling A (1995) Multiple polymorphisms, but no mutations, in the WAF1/CIP1 gene in human brain tumors. *Br J Cancer* **72**: 1230–1233.

Levine AJ (1997) *p53*, the cellular gatekeeper for growth and division. *Cell* **88**: 323–331.

Li Y, Jenkins CW, Nichols MA and Xiong Y (1994) Cell cycle expression and *p53* regulation of the cyclin-dependent kinase inhibitor p21. *Oncogene* **9**: 2261–2268.

Macleod KF, Sherry N, Hannon G, Beach D, Tokino T, Kinzler K, Vogelstein B and Jacks T (1995) Evidence for a *p53*-independent pathway for upregulation of SDI1/CIP1/WAF1/p21 RNA in human cells. *Genes Dev* **9**: 935–944.

Malkin D, Li FP, Strong LC, Fraumeni JrJF, Nelson CE, Kim DH, Kassel J, Gryka MA, Bishoff FZ, Tainsky MA and Friend SH (1990) Germ line *p53* mutations in familial syndrome of breast cancer, sarcomas and other neoplasms. *Science* **250**: 1233–1238.

Matsushita K, Kobayashi S, Kato M, Itoh Y, Okuyama K, Sakiyama S and Isono K (1996) Reduced messenger RNA expression level of p21 CIP1 in human colorectal carcinoma tissues and its association with *p53* gene mutation. *Int J Cancer* **69**: 259–264.

Mousses S, Ozcelik H, Lee PD, Malkin D, Bull SB and Andrulis IL (1995) Two variants of the CIP1/WAF1 gene occur together and are associated with human cancer. *Hum Mol Genet* **4**: 1089–1092.

Mousses S, McAuley L, Bell RS, Kandel R and Andrulis IL (1996) Molecular and immunohistochemical identification of *p53* alterations in bone and soft tissue sarcomas. *Mod Pathol* **9**: 1–6.

Özçelik H, Mousses S and Andrulis IL (1995) Low levels of expression of an inhibitor of cyclin-dependent kinases (CIP1/WAF1) in primary breast carcinomas with *p53* mutations. *Clinical Cancer Research* **1**: 907–912.

Paulovich AG, Toczyski DP and Hartwell H (1997) When checkpoints fail. *Cell* **88**: 315–321.

Reissmann PT, Simon MA, Lee WH and Slamon DJ (1989) Studies of the retinoblastoma gene in human sarcomas. *Oncogene* **4**: 839–843.

Shiohara M, El-Deiry WS, Wada M, Nakamaki T, Takeuchi S, Yang R, Chen DL, Vogelstein B and Koeffler HP (1994) Absence of WAF1 mutations in a variety of human malignancies. A mammalian cell cycle checkpoint pathway utilizing *p53* and *GADD45* is defective in ataxia-telangiectasia. *Blood* **84**: 3781–3784.

Sun Y, Hildesheim A, Li H, Li Y, Chen JY, Cheng YJ, Hayes RB, Rothman N, Bi WF and Cao Y (1995) No point mutation but a codon 31ser→arg polymorphism of the WAF-1/CIP-1/p21 tumor suppressor gene in nasopharyngeal carcinoma (NPC): the polymorphism distinguishes Caucasians from Chinese. *Cancer Epidemiol Biomarkers Prev* **4**: 261–267.

Taubert H, Meye A and Wurl P (1996) Prognosis is correlated with *p53* mutation type for soft tissue sarcoma patients. *Cancer Res* **56**: 4134–4136.

Waldman T, Kinzler KW and Vogelstein B p21 is necessary for the *p53*-mediated G1 arrest in human cancer cells (1995) *Cancer Res* **55**: 5187–5190.

Wan M, Cofer KF and Dubeau L (1996) WAF1/CIP1 structural abnormalities do not contribute to cell cycle deregulation in ovarian cancer. *Br J Cancer* **73**: 1398–1400.

Watanabe H, Fukuchi K, Takagi Y, Tomoyasu S, Tsuruoka N and Gomi K (1995) Molecular analysis of the WAF1 (p21) gene in diverse types of human tumors. *Biochem Biophys Acta* **1263**: 275–280.

Wunder JS, Czitrom AA, Kandel R and Andrulis IL (1991) Analysis of alterations in the retinoblastoma gene and tumor grade in bone and soft-tissue sarcomas. *J Natl Cancer Inst* **83**: 194–200.

Wunder JS, Eppert K, Burrow SR, Gokgoz N, Levine AJ, Bell RS and Andrulis IL (1999) Co-amplification and Overexpression of CDK4, SAS, and MDM2 in human parosteal osteosarcomas *Oncogene* **18**: 783–788.

Xiong Y, Hannon GJ, Zhang H, Casso D, Kobayashi R and Beach D (1993) p21 is a universal inhibitor of cyclin kinases. *Nature* **366**: 701–704.

*© 2001 Cancer Research Campaign*
